# Acceptability and feasibility of screening with a pediatric care provider-led social determinants of health identification tool

**DOI:** 10.1186/s12887-024-04759-2

**Published:** 2024-05-03

**Authors:** Alison Eyre, Janice Cohen, Sarah Funnell, Lynsey James, Sheena Guglani, Hounaida Abi Haidar, Lindy Samson, Michelle Ward, Radha Jetty, Megan Harrison, John S. Lyons, Leigh Fraser-Roberts, Susan Bennett, Douglas Archibald, Soha Khorsand, Tobey Audcent

**Affiliations:** 1 Department of Family Medicine, Faculty of Medicine, University of Ottawa, Ottawa, ON Canada; 2 Centretown Community Health Centre, Ottawa, ON Canada; 3 Department of Psychiatry, Faculty of Medicine, University of Ottawa, Ottawa, ON Canada; 4 Children’s Hospital of Eastern Ontario, Ottawa, ON Canada; 5 Department of Family Medicine, Faculty of Medicine, Queen’s University, Kingston, ON Canada; 6 Centre for Indigenous Health Research and Education, University of Ottawa, Ottawa, ON Canada; 7 Bruyère Research Institute, Ottawa, ON Canada; 8 Department of Paediatrics, Faculty of Medicine, University of Ottawa, Ottawa, ON Canada; 9 Children’s Hospital of Eastern Ontario Research Institute, Ottawa, ON Canada; 10 Centre for Innovation in Population Health, University of Kentucky, Lexington, KY USA; 11 Faculty of Medicine, University of Ottawa, Ottawa, ON Canada

**Keywords:** MeSH terms: Social Determinants of Health, MeSH terms: Pediatrics, Non-MeSH terms: Screening tool

## Abstract

**Background:**

Complex social determinants of health may not be easily recognized by health care providers and pose a unique challenge in the vulnerable pediatric population where patients may not be able to advocate for themselves. The goal of this study was to examine the acceptability and feasibility of health care providers using an integrated brief pediatric screening tool in primary care and hospital settings.

**Methods:**

The framework of the Child and Adolescent Needs and Strengths (CANS) and Pediatric Intermed tools was used to inform the selection of items for the 9-item Child and Adolescent Needs and Strengths-Pediatric Complexity Indicator (CANS-PCI). The tool consisted of three domains: biological, psychological, and social. Semi-structured interviews were conducted with health care providers in pediatric medical facilities in Ottawa, Canada. A low inference and iterative thematic synthesis approach was used to analyze the qualitative interview data specific to acceptability and feasibility.

**Results:**

Thirteen health care providers participated in interviews. Six overarching themes were identified: acceptability, logistics, feasibility, pros/cons, risk, and privacy. Overall, participants agreed that a routine, trained provider-led pediatric tool for the screening of social determinants of health is important (*n* = 10, 76.9%), acceptable (*n* = 11; 84.6%), and feasible (*n* = 7, 53.8%).

**Interpretation:**

Though the importance of social determinants of health are widely recognized, there are limited systematic methods of assessing, describing, and communicating amongst health care providers about the biomedical and psychosocial complexities of pediatric patients. Based on this study’s findings, implementation of a brief provider-led screening tool into pediatric care practices may contribute to this gap.

**Supplementary Information:**

The online version contains supplementary material available at 10.1186/s12887-024-04759-2.

## Introduction

Social Determinants of Health (SDH) are defined as “the conditions in which people are born, grow, live, work and age” [1, 2]. These conditions, such as social economic status (SES), education, adverse childhood experiences, and housing stability have a direct impact on the health and well-being of individuals [2–4]. SDH also impact patients’ abilities to equitably seek health care, adhere to treatments, and realize optimal medical outcomes [5, 6]. Despite their importance, assessing, communicating and integrating patients’ SDH remains uncommon in most health care settings.

The traditional approach to clinical care has involved a focus on biomedical factors (downstream approach), specifically, measurable somatic issues [7, 8]. This paradigm has shifted to the biopsychosocial model, which considers social and psychological factors in addition to biological (upstream determinants) [7, 8]. Complex SDH may not be easily recognized by health care providers (HCP) and can be difficult to describe in a clinically meaningful way [9]. In order to provide patients with the most effective health care, it is essential to find ways to measure and communicate a holistic view of patients’ needs to the entire health team in a way that is respectful of their unique perspectives and contexts. Multiple authors have suggested and/or developed stand-alone tools to screen for SDH [10–19], however, many of these tools are specific to certain age groups or clinical environments. There are few brief, versatile tools for use in the pediatric population in high volume settings.

Pediatrics poses a unique challenge as patients are particularly vulnerable and SDH among adult caregivers may directly or indirectly impact the child creating situations where developmental needs are not met, with potential lifelong consequences [4]. 

The aim of this study was to explore health care provider’s perceptions of the acceptability and feasibility of an integrated brief pediatric-specific tool to assess and describe SDH in community and hospital clinics.

## Methods

The study team included 15 active pediatric or family medicine HCPs and researchers. An initial review of the literature was conducted during the study development in 2015 and updated in 2020 with the support of a medical librarian. The search was conducted to identify existing pediatric-specific screening tools for social determinants of health. The searches included MeSH terms for child advocacy, social conditions, education and synonyms for children and pediatric patients within the MEDLINE (via EBSCO) database for a period of 10 years (2010–2020). A 10-year period was searched to identify records that were relatively recent (i.e., published in the prior decade). All relevant records from these searches were reviewed in full.

The CANS-PCI was developed using the framework of the Child and Adolescent Needs and Strengths (CANS) [20] and Pediatric Intermed [12] tools. A modified-Delphi process was used to create a condensed version. The CANS and Pediatric Intermed tools are based on the communimetric theory of measurement [20, 21]. This is a conceptual framework in which experts identify the key characteristics necessary for a sufficient clinical understanding of a concept and convert those characteristics into actionable indicator items. In other words, the pediatric experts meet and reach a consensus on the core elements of SDH as they present in pediatric care. The elements become items which are then placed into an action framework with four levels: 0 = No evidence, no need for action, 1 = watchful waiting/prevention, 2 = action, and 3 = immediate/intensive action.

The team developed an interview guide (Additional File 1) for semi-structured qualitative interviews [22], in order to examine health care provider perceptions about the feasibility and acceptability of an integrated brief pediatric-specific SDH identification tool. Our study used the CANS-Pediatric Complexity Indicator (CANS-PCI) which consisted of 9 items across three domains: biological, psychological and social (Additional File 2).

Participants were recruited at varied medical facilities in Ottawa, Ontario that serve pediatric populations face-to-face via staff at each institution who were emailed by the research team. Many of these facilities serve at-risk children and their families. Preliminary in-person meetings with members of the study team and one of the participating community health centers was conducted, in conjunction with a snowball sampling approach [23, 24]. The range of psychosocial and medical needs of the target population precluded the application of a pre-defined sampling frame and sampling approach. Therefore, recruitment was done using purposive convenience sampling [25]. 

Prior to providing written informed consent to participate in a 60-minute interview, participants were provided study information, including a list of the research team members, and the rationale for conducting the study. One of two research assistants (AR, SG) with training in qualitative methods conducted interviews with participants. These research staff members were not known to the interview participants. Participant recruitment and interviews took place between August 1, 2017 to May 31, 2018; interviews were conducted until no new themes were identified.

### Analysis of interviews

Interviews were recorded, transcribed verbatim, and imported into NVivo™ for analysis. Data analysis was done through a low inference approach [26] in an iterative fashion by the research assistant (SG) and one principal investigator (AE) using thematic synthesis [27]. These individuals were trained in qualitative methods, and the principal investigator was a family physician with clinical experience with vulnerable populations. Coding/recoding of the interviews were undertaken by these two team members, taking into consideration new data and emerging themes. Codes were aggregated into themes based on similarities and differences between codes. The study team reached consensus on the final themes. The following strategies were applied to validate the findings: [28] data collection and analysis was conducted in an iterative method with two analysts, and thick descriptions of each theme were generated.

Ethics approval was granted by local institutional research boards. Data use, recording and storage followed the rules and guidelines of three boards.

## Results

Out of 18 HCP who indicated that they were willing to be contacted, a total of 13 HCP participated in interviews (Table 1). The remaining 5 HCPs were unresponsive to follow-up attempts. Six overarching themes were identified most frequently, with several sub-themes (Table 2). Twelve (92.3%) expressed the need to understand SDH in their pediatric population and 11 (84.6%) were positive about the acceptability of using a tool to recognize and communicate about patients’ SDH.

**Table 1:** Participant demographics (*N* = 13)

Characteristic	*N* (%)
**Gender**	
Male	2 (15.4)
Female	11 (84.6)
**Number of years in practice**	
0–5	1 (7.7)
5–10	4 (30.8)
10+	8 (61.5)
**Time spent with client** (*N* = 12)	
20 min	6 (46.2)
60 min	3 (23.1)
Other	3 (23.1)
**Profession**	
Registered Nurse	1 (7.6)
Nurse Practitioner	4 (30.8)
Family Physician	3 (23.1)
Social Worker	1 (7.7)
Pediatrician	3 (23.1)
Surgeon	1 (7.7)
**Scope of practice**	
Community Health Centre	7 (53.8)
Adolescent Health Clinic	1 (7.7)
Academic Teaching Centre (secondary post)	2 (15.4)
Tertiary Hospital	5 (38.5)
Outpatient (secondary post)	2 (15.4)
**Population Served** ^**a**^	
Involvement in child welfare system	13 (100)
Poverty	13 (100)
First Nations Children	11 (84.6)
Metis Children	7 (53.8)
Inuit Children	8 (61.5)
Past or present immigration or refugee status	12 (92.3)
Complex medical needs	12 (92.3)
Caregiver substance use	12 (92.3)
Substance use/addiction	12 (92.3)
Inconsistent access to medical care	11 (84.6)
Legal issues/Criminal activity	12 (92.3)
Language barriers	13 (100)
Family/Domestic violence	12 (92.3)
Single parent household	13 (100)
Food insecurity	12 (92.3)
Inadequate housing	12 (92.3)
Caregiver medical problems	12 (92.3)

^a^ Participants were able to choose all that applied

**Table 2:** The most frequently occurring themes

Theme	Sub-themes
Acceptability	- Acceptability of a standardized tool - Aspects that make this more acceptable
Logistics	- Time - Computer versus Paper - Who administers it - When is it administered? - Training
Feasibility	
Pros/Cons	
Risk	- Judgement - General risks, Risk to Confidentiality - Privacy, Stigma - Past Trauma - Literacy, Outdated Information
Privacy	

Eight (61.5%) commented on acceptability of a standardised tool, if done carefully:

*“I think it would help […] sometimes because of the variety of different populations, [it would help] to have a clear picture of the SDH, [it] would be better to have the tools to better serve them”*. (Interviewee 10)

When asked *“how would you feel about asking questions from each of these categories to a patient”*, 12 (92%) agreed that they would be able to ask questions. However, 12 (92.3%) raised concerns about risks associated with such a tool (privacy, risk to confidentiality, judgement, and lack of health literacy) and ten (76.9%) were concerned about logistical issues (time to administer, who administers, method of administration, training) in their medical practices.

The majority of participants 10; (76.9%) agreed on the importance. Three essential elements were identified.

First, 7 (54%) identified that HCP would need specialized training in administering an SDH screening tool.

*“I think the tool should have come with some training like any history taking does. […] They look [like] straight content question[s], but they’re very loaded. All the families that we (serve), really are underserved”.* (Interviewee 11)

Second, 12 (91.3%) highlighted that the use of the tool should be sensitive to avoid judgement and stigma.

*“Some patients find it difficult or even offensive or, like, too much prying to ask things like income, or other determinants, like social determinants of health. So, I could see that it’s kind of a sensitive topic in a number of ways”.* (Interviewee 9)

Third, data collection should respect the relationship between HCP and patients. Eight (61.5%) spoke of the importance of the relationship in gathering sensitive data:

*“I’ve been in the business a long time. And every week, every day there’s some new tool for something in the last couple of years. And it’s to the point that, you know, I think that the talking to people and having a relationship with them, if we were to use all these tools would definitely suffer. So I am really on the fence about tools”*. (Interviewee 8)

*“… if a patient is asking, isn’t aware of what the link might be between some of these questions and they just seem invasive and personal that you risk jeopardizing a relationship that way. But I feel that that would be easier to deal with the right approach*”. (Interviewee 5)

As the tool was considered acceptable by the majority, 7 (53.8%) commented specifically on the feasibility of administering this tool.

Two of the HCP were already collecting SDH related data, albeit in an individualized fashion:

*“We do what’s called a HEADSS assessment, it’s a psychosocial component of our interview, so we look at the Home, Education, the HE, A is for activities, D for drugs, S for sexuality and S for suicide or depression”*. (Interviewee 6)

Potential risk was also identified as a main theme:

*“So what it is that I might be able to offer, and why that information might help with my diagnosis or my management plan. Just so that [patients] understand that it’s not prying and that I’m not asking for any form of judgment or anything like that”*. (Interviewee 9)

Seven (53.8%) commented on confidentiality:

*“Have it documented where it’s necessary and making sure that it’s confidential and still private. […] we are always reminded that patient confidentiality is key”.* (Interviewee 12)

*“I think once [patients] have that understanding that we’re not going to go and tell their parents, or whoever, their answers, there’s a lot more trust”.* (Interviewee 6)

Six (46%) commented on the risk of judgement:

*“As long as you try to do it in a non-judgmental way I guess it would be important to provide a rationale on every question or a little caveat explaining where this question comes from, like what’s the benefit of it”.* (Interviewee 4)

Five (38%) commented on potential stigma:

*“The only thing is hopefully people don’t feel stigmatized. That’s the only harm that I could see”*. (Interviewee 10)

Five (38%) were concerned about privacy:

*“Even though the chart is open for everyone to see, we’re not pointing it out all [to] the other providers. […] sometimes there’s a reason a more sensitive topic is just being discussed with one provider”*. (Interviewee 9)

Six (46%) highlighted time as a concern:

*“[Health care providers] will not be having the time to ask all of this. So it would be unrealistic to think in a busy surgical clinic that they’re going to be…It’s different than the pediatric origin of practice”*. (Interviewee 13)

Three (23%) commented on literacy:

*“… I guess it’s with experience where you sort of, you might have a hunch about somebody’s ability to…navigate the system based on their literacy”*. (Interviewee 5)

And three (23%) were concerned that the information might become outdated but remain on the patient’s file:

*“They saw somebody at some point that said they had an addiction or that they didn’t finish University [.] and that’s outdated. […] This is 10-year-old information you’re working with and that addiction could be well into recovery and they’re being stigmatized”*. (Interviewee 7)

Overall, the HCPs in this study highlighted the impact of SDH screening despite noting some potential risks, and most described the use of a short tool as both acceptable and feasible.

## Interpretation

Despite the recognition of the importance of SDH on health care delivery and outcomes and a proliferation of new tools for screening, more study is needed to inform best practices for HCPs to assess and communicate SDH. The Canadian Medical Association has called for increased recognition of the critical impact of SDH, identified as integral to improving the health of Canadians [29, 30]. In 2016, the Centre for Effective Practice endorsed the use of screening tools and a tool kit, adaptable to various Electronic Health Records used in Canada to support taking SDH into account in primary care [31]. The American Academy of Pediatrics recommends systems that both identify developmental issues and provide links to supportive community services [32]. Currently, there is no universally accepted systematic way of assessing, describing and communicating pediatric patients’ combined biomedical and psychosocial needs and their complexity in order to facilitate access to services and resources.

Patients’ complex narratives are difficult to quantify, and although there are several existing tools that include SDH items, they tend to be specific to either a particular age group or clinical environment, and thus are not generalizable to a variety of settings. In a 2019 systematic review, Sokol and colleagues identified 11 unique screening tools published in the last 12 years with the primary aim of supporting the recommendations to identify vulnerable children [33]. However, most such tools screened for specific issues only (such as finance or health literacy) or were specific to a particular age group or clinical environment. In addition, there was limited evaluation of the tools’ psychometric properties.

Several authors have looked at the feasibility and acceptability of implementing them [13, 15, 17, 34]. Similar to the findings of this body of research, we found that logistical matters and capacity were common themes. The key is keeping it simple and flexible and not add to the documentation burden of the work [10, 16, 35, 36]. 

In-line with the above-mentioned literature, HCP in this study expressed a good understanding of the impact of SDH on their populations and interest in collecting that data in a way that would positively impact patients. However, despite their roles working with populations at risk, only two HCP indicated routinely assessing for SDH.

The participants in this study endorsed that training of staff in administration of any tool would be key to minimizing stigma and bias, much in line with the literature [35, 37]. A 25-day feasibility trial incorporating an SDH screening tool in the Emergency Department concluded that the main challenges were provider discomfort screening based on patient appearance, asking stigmatizing questions, and lack of clarity regarding the screening’s purpose [18]. 

HCP in this study were in agreement across several key themes that were caveats for implementation:

1. How would the tool be integrated into the visit to avoid risk of bias or stigma?
2. How to respect the needs, languages and cultures of different group?
3. What training would be provided to the interviewer to ensure the questions were asked in a patient and caregiver centered way?
4. How would potential risks of unintended harms in the form of perpetuation of stigma, racism, bias or discrimination be addressed?

Overall, this work demonstrates that the interactions between SDH and pediatric health challenges are complex. There are distal determinants of health from which intermediate and proximal determinants of health flow. This complexity is further compounded by parent and guardian social and economic situation, including intergenerational effects of trauma [38]. 

### Limitations

This study involved a small number of HCP from a specific health care system and the interviews were conducted pre-pandemic. A family/patient research advisory committee was launched at our center after the start of the study and would be a valuable partner in research of this kind in the future. This highlights the role of newer methodologies to engage patients as partners in research from its inception [39]. While our study used an established framework, the reliability and validity of the CANS-PCI as a screening tool has not been established. A shortened tool enhances feasibility but may also risk oversimplification of complex societal or contextual contributors. More study is needed to identify how patients’ needs should be recorded and shared.

### Future directions in the area of study

Healing-centred approaches highlight strengths and cultural contributions to well-being [40]. This study did not specifically examine how practitioners can implement a healing-centred approach to care plans and communication. There is more work that is needed to understand how using such strategies can mitigate equity gaps experienced by those with complex SDH concerns rooted in racism, bias, stigma, or trauma and how best to communicate about these issues with patients and families.

## Conclusions

Although the importance of SDH is widely recognized and there are pediatric-specific SDH screening tools available, there is no existing systematic way of assessing, describing and communicating between HCP about pediatric patients’ combined biomedical and psychosocial complexities. It appears from this study, a short SDH screening tool (such as the CANS-PCI) may be able to contribute to this gap. By using the communimetric framework [20, 21], this project builds on an established measurement platform and a commonly used measurement framework for child-serving systems in North America.

Overall, HCP in our study agreed that a routine, brief integrated pediatric specific tool to screen for SDH is important and would be both acceptable and feasible. Ultimately, this small study contributes to the growing body of evidence that HCP understand the importance of SDH to their patients and would be willing to integrate this into their practice. What matters more than “what” tool is used is “how” it is used, and by “whom”.

Further research needs to be conducted to: (1) include different contexts and the point of view of the patients and their HCP, (2) answer the larger question of whether routine use of a screening tool by HCP will facilitate patient access to services and improve health outcomes, and (3) incorporate best practices/standards about how such tools should be used, and by whom and how the findings should be communicated with patients and families.

### Electronic supplementary material

Below is the link to the electronic supplementary material.

Additional File 1: Questions for Cognitive Interview

Additional File 2 ? CANS-PCI Tool. Child/Youth and Adolescent Needs and Strengths (CANS?) PEDIATRIC COMPLEXITY INDICATOR VERSION; CANS-PCI? MANUAL

Supplementary Material 3

## Data Availability

The de-identified dataset used and analyzed during the current study are available from the corresponding author on reasonable request. To preserve the anonymity of the interviewees, the transcribed interviews are not available for sharing.
