# Supplementary material for: Acceptability and feasibility of screening with a pediatric care provider-led social determinants of health identification tool
Source: BMC Pediatr. 2024 May 3;24:300. doi: 10.1186/s12887-024-04759-2 (PMC11067211; doi:10.1186/s12887-024-04759-2)
Supplement: Supplementary file 1 — Additional File 1: Questions for Cognitive Interview [file 12887_2024_4759_MOESM1_ESM.pdf]

7/9/13

Child/Youth and Adolescent Needs and Strengths

(CANS©)

PEDIATRIC COMPLEXITY INDICATOR VERSION

CANS-PCI©

MANUAL

Praed Foundation

Copyright 1999, 2013

A large number of individuals have collaborated in the development of the CANS-PCI. Along with the CANS versions for developmental disabilities, juvenile justice, and child/youth welfare, this information integration tool is designed to support individual case planning and the planning and evaluation of service systems. The CANS-PCI is an open domain tool for use in service delivery systems that address the health of children, adolescents and their families. The copyright is held by the Praed Foundation to ensure that it remains free to use. For specific permission to use please contact the Praed Foundation. For more information on this tool, please contact:

Tobey Audcent, MD, Janice Cohen, PHD, Megan Harrison, MD,
Radha Jetty, MD, Lindy Samson, MD, Michelle Ward, MD, Josee Blackburn, MSW
Jama Watt
Children's Hospital of Eastern Ontario
401 Smyth Road
Ottawa, ON

Allison Eyre, MD, Linsey James, Sarah Funnell, MD
Centertown Community Health Centre
420 Cooper street
Ottawa, ON

John S. Lyons, Ph.D.
jlyons@uottawa.ca

Praed Foundation
550 N. Kingsbury Street, Suite 101
Chicago, IL 60654
praedfoundation@yahoo.com
www.praedfoundation.org

CANS-Pediatric Complexity Indicators
CANS-PCI

Child's Name: ________________________________ Date: ________________ ID: ______________

Date of Birth __________________ Caregiver: ☐ Parent
☐ Other Relative
☐ Foster Care
☐ Other ______________________________

	**0**	**1**	**2**	**3**	**Comment** (**2 or 3 ratings**)
Medical/Diagnostic Complexity	☐	☐	☐	☐	__________________________
Behavioral/Emotional Needs	☐	☐	☐	☐	___________________________
Developmental Needs	☐	☐	☐	☐	___________________________
Caregiver Needs	☐	☐	☐	☐	___________________________
Social Complexity	☐	☐	☐	☐	___________________________
Educational Status	☐	☐	☐	☐	___________________________
Organizational Complexity	☐	☐	☐	☐	___________________________
Hospitalizations in past year	☐	☐	☐	☐	___________________________

	**Score**	**Recommendations**
Total Number of 2 or 3 ratings: ____	0 to 1	☐ standard care
	2	☐ simple care coordination
	3 to 4	☐ enhanced care coordination
	5 and up	☐ intensive care coordination
Total Number of 1 ratings: ____	0 to 1	☐ standard care
	2 or higher	☐ diagnostic teaming/coordination

INTRODUCTION

The **CANS** is a multiple purpose information integration tool that is designed to be the output of an assessment process. The purpose of the **CANS** is to accurately represent the shared vision of the child/youth serving system—children, youth, and families. As such, completion of the **CANS** is accomplished in order to allow for the effective communication of this shared vision for use at all levels of the system. Since its primary purpose is communication, the **CANS** is designed based on communication theory rather than the psychometric theories that have influenced most measurement development. There are six key principles of a communimetric measure that apply to understanding the **CANS**.

<u>Six Key Principles of the CANS</u>

1. Items were selected because they are each relevant to the explicit purpose of the version. An item exists because it might lead you down a different pathway in terms of planning actions.
2. Each item uses a 4-level rating system. Those levels are designed to translate immediately into action levels. Different action levels exist for needs and strengths.
3. Rating should describe the child/youth, not the child/youth in care. If an intervention is present that is masking a need but must stay in place, this should be factored into the rating consideration and would result in a rating of an "actionable" need (i.e. "2" or "3").
4. Culture and development should be considered prior to establishing the action levels. Cultural sensitivity involves considering whether cultural factors are influencing the expression of needs and strengths. Ratings should be completed considering the child/youth's developmental and/or chronological age depending on the item.
5. The ratings are generally "agnostic as to etiology". In other words this is a descriptive tool; it is about the "what" not the "why".
6. A 30-day window is used for ratings in order to make sure assessments stay "fresh" and relevant to the child/youth's present circumstances. However, the action levels can be used to over-ride the 30-day rating period.

<u>Action Levels for "Need" Items</u>

0 – No Evidence of Need – This rating indicates that there is no reason to believe that a particular need exists. Based on current assessment information there is no reason to assume this is a need.

1 - Watchful Waiting/Prevention – This level of rating indicates that you need to keep an eye on this area or think about purposing further assessment—history, suspicion or contention are reasons for this rating.

2 - Action Needed – This level of rating implies that something must be done to address the identified need. The need is sufficiently problematic, that it is interfering in the child/youth's or family's life in a notable way.

3 - Immediate/Intensive Action Needed – This level rating indicates a need that requires immediate or intensive effort to address. Dangerous or disabling levels of needs are rated with this level.

CANS-Pediatric Complexity Indicators (CANS-PCI)

Item Anchored Definitions of Action Levels

BIOLOGICAL DOMAIN

Check	**Medical/Diagnostic Complexity**
0	The child's/youth's medical diagnoses are clear and there is no doubt as to the correct diagnoses; symptom presentation is clear.
1	Although there is some confidence in the accuracy of child's/youth's diagnoses, there also exists sufficient complexity in the child's/youth's symptom presentation to raise concerns that the diagnoses may not be accurate.
2	There is substantial concern about the accuracy of the child's/youth's medical diagnoses due to the complexity of symptom presentation.
3	It is currently not possible to accurately diagnose the child's/youth's medical condition(s).

Check	**Physical Symptom Severity** *Please rate the highest level from the **past 30 days***
0	No physical symptoms or symptoms resolve with treatment.
1	Mild symptoms which do not interfere with current functioning.
2	Moderate symptoms, which interfere with current functioning.
3	Severe symptoms leading to an inability to perform most functional activities.

PSYCHOLOGICAL DOMAIN

Check	**Developmental Needs/Cognitive Intellectual Development** *Please rate the highest level from the **past 30 days***
0	No evidence of any current developmental problems.
1	Mild problems with mild developmental delays. Parents or teachers may express some concern.
2	Developmental problems are notably interfering with child's functioning in at least one domain (e.g. home, school)
3	Child has a severe developmental problems. Child is completely disabled or unable to care for self in a developmentally appropriate manner.

Check	**Emotional/Behavioral Needs** *Please rate the highest level from the **past 30 days***
0	No evidence of any current emotional problems (e.g. depression, anxiety, traumatic response) or behavioral (e.g. opposition to authority, delinquency).
1	Mild problems with emotions or behaviors. Parents or teachers may express some concern. Problems do not appear to have notable impact on child's functioning at this time. Reduced coping skills.
2	Emotional or behavioral problems are notably interfering with child's functioning in at least one domain (e.g. home, school).
3	Child has a severe level of emotional or behavioral problems. Child is completely disabled or unable to care for self in a developmentally appropriate manner.

Check	**Child/Caregiver Barriers to Coping**
0	Child and/or Caregiver able to adapt well to life stresses and health circumstances. Flexible.
1	Reduced coping skills. Child/youth and /or caregiver may have difficulties adapting to life stresses/changes, and health circumstances. No long-term difficulties.
2	Impaired coping skills, child and/or caregiver has significant difficulty adjusting to stresses and changes in his or her life. Potential long term difficulties.
3	No evidence of any coping skills. Inability to adapt to life stresses, changes, or to deal with unresolved feelings/emotions. Immediate risk.

SOCIAL DOMAIN

Check	**School Functioning and Educational Needs** *Please rate the highest level from the* ***past 30 days***
0	Performing well in school with good achievement, attendance and behavior. No academic concerns.
1	Performing adequately in school although some achievement, attendance or behaviour problems which have not dramatically affected the child's educational progress. Academic concerns are being addressed adequately.
2	Experiencing moderate problems with school achievement, attendance and/or behaviour that are currently interfering with the child's educational progress. Academic concerns are only partially addressed.
3	Experiencing severe problems with school achievement, attendance and/or behaviour, preventing the child from educational progress consistent with their current developmental status. Academic concerns are not currently being addressed.

Check	**Caregiver (Parent) Health & Function** *Please rate the highest level from the* ***past 30 days***
0	No evidence of any current physical and /or mental health conditions including substance use-related or developmental problems among individuals responsible for providing parenting/care to the child.
1	Physical and /or mental health conditions including substance use-related or developmental problems present in one or more caregiver, which do not impact parenting.
2	Physical and /or mental health conditions including substance use-related or developmental problems interfere with the caregiver(s) ability to take care of the child/youth.
3	Physical and /or mental health conditions, including substance use-related or developmental problems present in one or more caregivers that prevent effective parenting and/or create a dangerous situation for the child/youth.

Check	**Social-Environmental Complexity & Residential Stability** *Please rate the highest level from the* ***past 30 days***
0	No evidence of any social (financial ,environmental)factors that influence the child's health and wellbeing. Stable housing and financial support for personal growth needs.
1	Some social factors are suspected that might impact the child's health or mild social challenges influence the child or family's ability to address health needs. Mild stress with multiple moves, school changes, financial issues.
2	Social problems are notably interfering with child's health or functioning in at least one domain (e.g. home, school).Moderate stress with unstable housing and/or living situation support (e.g. living in a shelter, poor nutrition, change of current living situation is required, unemployment).
3	Severe level of social factors that dramatically interfere with the child's health or wellbeing. Factors such as severe poverty, homelessness, or discrimination etc are having a significant effect on the child and/or family. Child youth is malnourished or in a dangerous environment; immediate change is necessary.

Check	**Organizational Complexity**
0	All medical care is provided by a single health care professional.
1	Child's/Youth's care is generally provided by a coordinated team of professionals who all work for the same organization.
2	Child's/Youth's care requires collaboration of multiple professionals who work for more than one organization but current communication and coordination is effective.
3	Child's/Youth's medical care requires the collaboration of multiple professionals who work for more than one organization and problems currently exist in communication among these professionals.
