# Supplementary material for: Acceptability and feasibility of screening with a pediatric care provider-led social determinants of health identification tool
Source: BMC Pediatr. 2024 May 3;24:300. doi: 10.1186/s12887-024-04759-2 (PMC11067211; doi:10.1186/s12887-024-04759-2)
Supplement: Supplementary file 2 — Additional File 2 ? CANS-PCI Tool. Child/Youth and Adolescent Needs and Strengths (CANS?) PEDIATRIC COMPLEXITY INDICATOR VERSION; CANS-PCI? MANUAL [file 12887_2024_4759_MOESM2_ESM.docx]

# Consolidated criteria for Reporting Qualitative Research (COREQ) Checklist (Tong et al., 2007)

| **Item** | **Guide questions/description** | **Response and page number referenced** |
| --- | --- | --- |
| *Domain 1: Research team and reflexivity* | |  |
| 1. Interviewer/ facilitator | Which author/s conducted the interview or focus group? | Page 6:  “One of two researchers (AR, SG) with training in qualitative methods conducted interviews with participants.” |
| 1. Credentials | What were the researcher’s credentials? e.g. PhD, MD | Researcher credentials are provided in the author list (page 1-3). |
| 1. Occupation | What was their occupation at the time of the study? | The research assistant completing data collection and analysis was employed for the study. The principal investigator who also contributed to data collection and analysis is a physician. |
| 1. Gender | Was the researcher male or female? | This detail was not included in the manuscript as the interviewer’s gender did not have a bearing on the content of interviews. |
| 1. Experience and training | What experience or training did the researcher have? | Page 6:  “One of two researchers (AR, SG) with training in qualitative methods conducted interviews with participants.” |
| 1. Relationship established | Was a relationship established prior to study commencement? | N/A |
| 1. Participant knowledge of the interviewer | What did the participants know about the researcher? e.g. personal goals, reasons for doing the research | Page 6:  “Prior to providing consent to participate in an interview, participants were provided study information, including a list of the research team members, and the rationale for conducting the study.” |
| 1. Interviewer characteristics | What characteristics were reported about the interviewer/facilitator? e.g. Bias, assumptions, reasons and interests in the research topic | Page 6:  “Prior to providing consent to participate in an interview, participants were provided study information, including a list of the research team members, and the rationale for conducting the study.” |
| *Domain 2: Study design* | |  |
| 1. Methodological orientation and Theory | What methodological orientation was stated to underpin the study? e.g. grounded theory, discourse analysis, ethnography, phenomenology, content analysis | Page 7:  “Data analysis was done through a low inference approach^22^ in an iterative fashion by the research assistant and one principal investigator using thematic synthesis.^23^” |
| 1. Sampling | How were participants selected? e.g. purposive, convenience, consecutive, snowball | Page 6:  “Preliminary in-person meetings with members of the study team and one of the participating community health centers was conducted, in conjunction with a snowball sampling approach.^19,20^ The range of psychosocial and medical needs of the target population precluded the application of a pre-defined sampling frame and sampling approach. Therefore, recruitment was done by purposive convenience sampling.^21^” |
| 1. Method of approach | How were participants approached? e.g. face-to-face, telephone, mail, email | Page 6:  “Participants were recruited at varied medical facilities in Ottawa, Ontario that serve pediatric populations face-to-face via staff at each institution who were emailed by the research team.” |
| 1. Sample size | How many participants were in the study? | Page 7:  “Out of 18 HCP who indicated that they were willing to be contacted, a total of 13 HCP participated in interviews (Table 1).” |
| 1. Non-participation | How many people refused to participate or dropped out? Reasons? | Page 7:  “Out of 18 HCP who indicated that they were willing to be contacted, a total of 13 HCP participated in interviews (Table 1). The remaining 5 HCPs were unresponsive to follow-up attempts.” |
| 1. Setting of data collection | Where was the data collected? e.g. home, clinic, workplace | Interviews were conducted via telephone, therefore, participants were able to participate from a location of their choosing. |
| 1. Presence of non-participants | Was anyone else present besides the participants and researchers? | Page 6:  “Interviews led by 1/2 team members were recorded and transcribed verbatim.” |
| 1. Description of sample | What are the important characteristics of the sample? e.g. demographic data, date | Participant demographic characteristics are presented in Table 1 (page 22-23). |
| 1. Interview guide | Were questions, prompts, guides provided by the authors? Was it pilot tested? | Page 6:  “The team developed an interview guide (Additional File 1) for semi-structured qualitative interviews,^18^ in order to examine HCP perceptions about the feasibility and acceptability of an integrated brief pediatric specific SDH identification tool, the CANS- Pediatric Complexity Indicator (CANS-PCI).” |
| 1. Repeat interviews | Were repeat interviews carried out? If yes, how many? | N/A |
| 1. Audio/visual recording | Did the research use audio or visual recording to collect the data? | Page 7:  “Interviews were recorded, transcribed verbatim, and imported into NVivo^TM^ for analysis.” |
| 1. Field notes | Were field notes made during and/or after the interview or focus group? | N/A |
| 1. Duration | What was the duration of the interviews or focus group? | Page 6:  “Prior to providing consent to participate in a 60-minute interview...” |
| 1. Data saturation | Was data saturation discussed? | Page 6:  “Participant recruitment continued and interviews were conducted until no new themes were identified.” |
| 1. Transcripts returned | Were transcripts returned to participants for comment and/or correction? | Transcripts were not returned to participants. Instead, the trustworthiness of the data collection and analysis procedures was ensured by conducting data collection and analysis in an iterative way with two analysts, and thick descriptions of each theme were generated. See page 6. |
| *Domain 3: Analysis and findings* | |  |
| 1. Number of data coders | How many data coders coded the data? | Page 7:  “Coding/recoding of the interviews were undertaken by these two team members, taking into consideration new data and emerging themes.” |
| 1. Description of the coding tree | Did authors provide a description of the coding tree? | Page 7:  “Coding/recoding of the interviews were undertaken by these two team members, taking into consideration new data and emerging themes. Codes were aggregated into themes based on similarities and differences between codes.” |
| 1. Derivation of themes | Were themes identified in advance or derived from the data? | Page 7:  “Data analysis was done through a low inference approach^22^ in an iterative fashion by the research assistant (SG) and one principal investigator (AE) using thematic synthesis.^23^” |
| 1. Software | What software, if applicable, was used to manage the data? | Page 7:  “Interviews were recorded, transcribed verbatim, and imported into NVivo^TM^ for analysis.” |
| 1. Participant checking | Did participants provide feedback on the findings? | N/A |
| 1. Quotations presented | Were participant quotations presented to illustrate the themes / findings? Was each  quotation identified? e.g. participant number | Page 9-12:  Participant quotations are presented in text to illustrate the themes. Quotations are identified by participant number. |
| 1. Data and findings consistent | Was there consistency between the data presented and the findings? | The findings were derived from the data using a low inference approach (see page 7), and therefore are consistent with the data presented. |
| 1. Clarity of major themes | Were major themes clearly presented in the findings? | Major themes are clearly presented in the body of the paper where representative participant quotations are provided for each theme. Table 2 (page 8) also lays out each theme and sub-theme. |
| 1. Clarity of minor themes | Is there a description of diverse cases or discussion of minor themes? | Sub-themes are clearly presented in the body of the paper where representative participant quotations are provided for each theme. Table 2 (page 8) also lays out each theme and sub-theme. |

**From:** Tong A, Sainsbury P, Craig J. Consolidated criteria for reporting qualitative research (COREQ): a 32-item checklist for interviews and focus groups. International Journal for Quality in Health Care. 2007. Volume 19, Number 6: pp. 349 – 357
