# Supplementary material for: Acceptability and feasibility of screening with a pediatric care provider-led social determinants of health identification tool
Source: BMC Pediatr. 2024 May 3;24:300. doi: 10.1186/s12887-024-04759-2 (PMC11067211; doi:10.1186/s12887-024-04759-2)
Supplement: Supplementary file 3 — Supplementary Material 3 [file 12887_2024_4759_MOESM3_ESM.pdf]

Additional File 1

Semi-Structured Interview Guide (Phase 1) and Cognitive Interview Questions (Phase 2)

Preamble

The health of children and families is influenced by the living conditions they experience. These conditions have come to be known as the social determinants of health. These aspects can include education, food and housing, and may be outside of our control.

Health care providers have difficulty describing and communicating these factors. This can lead to difficulties in accessing services and difficulty following a health care provider's advice.

Our group of care providers from both the community and CHEO want to develop a tool to describe these factors and their impacts on our patients. Additionally, professional involved in a child's care would be able to communicate about these factors and ensure that we can manage the healthcare needs of patients appropriately.

In order to help us develop this tool, we are asking you to tell us about your experiences so we can identify the key barriers that people face and how we might best help them overcome these barriers.

Consent

- Have you had a copy of the information sheet and consent form?
- Do you have any questions? [answer any questions]
- Are you happy to proceed with the interview?
- Are you okay with me recording the interview?

Phase 1: Semi–Structured Interview

PART 1:

- Do you feel that a health care provider could use a standard set of questions to understand a patient's medical and social needs?
    - Probe: How do you think this could help?

- Are there any reasons you can think of why this might not be good or helpful?
- What do you feel would be the best way to gather this information from your patients?
- How would you feel about using a standard set of questions with patients in this way?
- How well do you think you would be able to answer these questions about a patient and his or her family?
- If you think it would be hard to answer these questions, what would make it easier?
- Do you think this process would be helpful for the patient and his or her family?
    - Probe: If yes, in what ways might this be useful? If not, why not?
    - Probe: Can you think of any potential risks or harms in doing this?
- Should the standard set of questions include a question answered the patient?
    - How would you feel about asking patients to do this?
    - Do you think it would be a helpful part of the process?
    - If yes, what would be the most important thing to ask the patient about his or her situation?

PART 2:

An existing tool identified 8 areas that are important and I'd like to ask you:

- How important you feel these are to a child's health needs?
- If you feel we have missed anything important, what else would you ask?
- How would you feel about asking questions about each of these aspects?

(Go through each one individually using the definition provided to explain what we mean)

Medical/Diagnostic Complexity: This describes the complexity of accurately diagnosing the child's medical condition based on available information.

Emotional/Behavioral Needs: This describes whether there are any emotional or behavioral challenges. Emotional challenges could include depression or anxiety, for example. Behavioral challenges could include oppositional or impulsive behavior.

Developmental Needs: This describes the presence of any intellectual or developmental challenges. For example, does the child have developmental delay?

Educational Status: This describes whether the child has any special educational needs.

Caregiver Needs: This would describe whether there were any additional needs for the caregiver(s) of the child.

Social/Cultural Complexity: This would include any other social factors such as language barriers, financial difficulties, or other social challenges.

Organizational Complexity: This refers to the number of health care professionals necessary to provide comprehensive and effective care to the child and family AND the current status of communication and collaboration among health care professionals in situations where multiple are involved.

Number of Hospitalizations in Past year: (any reason): This item counts the number of hospital admissions in the past 12 months regardless of the reason for the hospital admissions

Demographic Info for Health Care Providers:

With what gender do you identify?

Male	Female
☐	☐

Number of years in practice?

<2 years

5-10 years

>10 years

What is your professional role?

Pediatrician ☐

General practitioner ☐

Nurse ☐

Nurse Practitioner ☐

Social worker ☐

Other (please specify): ______________________ ☐

1.1 In what setting do you practice (please select as many that are relevant)?

Community healthcare center ☐

Outpatient clinic ☐

Private Community Practice ☐

Urgent Care clinic ☐

Emergency ☐

Non-academic hospital ☐

Academic teaching center ☐

Other (please specify): ______________________ ☐

1.2 What are the vulnerable population demographics in your practice? Select all that apply.

Involvement in child welfare system ☐

Poverty ☐

First Nations, Métis or Inuit children ☐

Past or present immigrant or refugee status ☐

Complex medical needs ☐

Caregiver substance abuse ☐

Inconsistent access to medical care ☐

Legal issues/criminal activity ☐

Language barriers ☐

Family/domestic violence ☐

Single parent household ☐

Food insecurity ☐

Inadequate housing ☐

Caregiver medical problems ☐

Other (please specify): ______________________ ☐

1.3 What would you estimate is your average time allotted for an appointment?

____

Phase 2: Cognitive (‘Think-aloud’) Interview Questions

Preamble

The health of children and families is influenced by the living conditions they experience. These conditions have come to be known as the social determinants of health. These aspects can include education, food and housing, and may be outside of our control.

Health care providers have difficulty describing and communicating these factors. This can lead to difficulties in accessing services and difficulty following a health care provider’s advice.

Our group of care providers from both CHEO and the Bruyère wants to develop a tool to describe and communicate about how these factors are impacting our patients and ensure that we can manage the healthcare needs of patients appropriately.

In order to help us develop this tool, we are asking you to tell us about your experiences so we can identify the key barriers that people face and how we might best help them overcome these barriers.

Consent

- Have you had a copy of the information sheet and consent form?
- Do you have any questions? [answer any questions]
- Are you happy to proceed with the interview?
- Are you okay with me recording the interview?

Cognitive (‘Think-aloud’) Interview

For the purpose of training the subject to perform a ‘think-aloud’ interview, the interviewer will ask the participant the following:

“Try to visualize the place where you live, and think about how many windows are in that place. As you count up the windows, tell me what you are seeing and thinking about.”

1. Can you explain to me in your own words what this item is asking you?
2. What does the term [*insert key term from an item*] mean to you in this item?
3. Can you explain to me why you chose the answer that you did?
4. Do you feel that the response options allowed you to answer the item appropriately? Explain.
5. Do you find the item easy or difficult to answer? Explain.
6. Do you feel this item should be included on the CANS-PCI? Explain.

General probes:

- “Can you tell me about how you came to choose this rating?”
- “Was that easy or hard to answer?”
- “I noticed that you hesitated– tell me what you were thinking.”
